# D-Dimer as a Sensitive Biomarker of Survival Rate in Patients with COVID-19

**DOI:** 10.5152/eurasianjmed.2022.21145

**Published:** 2022-10-01

**Authors:** Fadhilatul Hilda, Phey Liana, Awan Nurtjahyo, Harun Hudari, Nurmalia Purnama Sari, Tungki Pratama Umar, Chris Alberto Amin, Astari Rahayu Afifah

**Affiliations:** 1 Medical Profession Program, Universitas Sriwijaya Faculty of Medicine, Palembang, Indonesia; 2 Department of Clinical Pathology, Universitas Sriwijaya – Mohammad Hoesin General Hospital, Palembang, Indonesia; 3 Biomedicine Doctoral Program, Faculty of Medicine, Universitas Sriwijaya, Palembang, Indonesia; 4 Department of Obstetrics and Gynecology, Universitas Sriwijaya – Mohammad Hoesin General Hospital, Palembang, Indonesia; 5 Department of Internal Medicine, Universitas Sriwijaya – Mohammad Hoesin General Hospital, Palembang, Indonesia

**Keywords:** COVID-19, D-dimer levels, mortality

## Abstract

**Objective:**

The global case fatality rate of coronavirus disease 2019 is 2.16% as announced by the World Health Organization. In Indonesia, according to the Ministry of Health, the number is even higher, reaching a 2.8% case fatality rate. D-dimer levels were found to affect coronavirus disease 2019 patient’s survival in several studies. The study aimed to determine whether the amount of D-dimer predicted survival in coronavirus disease 2019 patients.

**Materials and Methods:**

This research was performed in a retrospective cohort design and used survival analysis. From March 1, 2020, to August 31, 2020, the samples were collected from polymerase chain reaction-confirmed coronavirus disease 2019 patients at Mohammad Hoesin General Hospital in Palembang, South Sumatera, Indonesia. We used electronic medical records to obtain demographic (age and gender), coexisting condition, laboratory (coagulation and hematologic test), and outcome (non-survivors or survivors) data. The chi-square and Mann–Whitney tests were used to evaluate the results. The Kaplan–Meier method and the Mantel–Haenszel log-rank test were used to examine D-dimer levels and patient outcomes. Youden index was calculated to determine the optimal cut-off value of D-dimer.

**Results:**

There were 52 non-survivors and 235 survivors among the 287 patients who met the inclusion criterion. Non-survivors had D-dimer levels of more than 1.49 mg/L in 82.69% of cases. Males had lower cut-off compared to females (>1.49 mg/L vs. >2.2 mg/L). The researchers discovered a highly significant correlation between D-dimer levels and coronavirus disease 2019 mortality (*P* = .001). The c-index analysis showed that D-dimer (0.79, 95% CI: 0.73-0.83) ability for mortality prediction was the second-best compared with other laboratory markers.

**Conclusion:**

D-dimer can be used as a predictor of coronavirus disease 2019 in-hospital mortality for early identification of coagulopathy.

**Main Points**

- Some demographic data including age and gender were considered as the most essential factors in predicting patients’ survival.
- The proposed cut-off for D-dimer in predicting patient outcome was 1.49 mg/L
- The use of D-dimer as a predictive measurement of patient death has 82.69% sensitivity and 68.09% specificity.
- Higher D-dimer (>1.49 mg/L) was associated with a lower 30-day survival rate than the lower D-dimer groups (8 days difference).
- D-dimer was the second-best laboratory marker for the mortality prediction of coronavirus disease 2019 patients.

## Introduction

Coronavirus disease 2019 (COVID-19) is an infectious disease caused by the severe acute respiratory syndrome coronavirus-2 (SARS-CoV-2), a novel member of group 2B β-coronavirus.^1^ The World Health Organization recorded 100 819 363 confirmed COVID-19 cases and 2 176 159 deaths worldwide as of January 29, 2021. Meanwhile, in Indonesia, since the first report of the case, there have been 1 051 795 confirmed COVID-19 cases and 29 518 deaths (case fatality rate: 2.8%) at the same date.^2^

The real-time reverse transcription-polymerase chain reaction (RT-PCR) test has become the gold standard for diagnosing COVID-19.^3^ However, several other laboratory markers, such as complete blood counts, hemostasis parameters, and inflammatory markers, are thought to be important, particularly as predictors of disease severity and prognosis.^4^

Increased D-dimers are one of the most common laboratory results found in COVID-19 patients.^5,6^ However, in previous studies, the D-dimer cut-off point was found to vary. It is most widely recognized as greater than 2.00 mg/L,^7-9^ but one study found that a cut-off of 1.00 mg/L was adequate as a predictive biomarker.^10^ The International Society of Thrombosis and Haemostasis recommends using the D-dimer value of 3-4 times higher than the initial level upon hospital admission as the cut-off^11^ and carrying out treatment with close monitoring.^7^ This phenomenon is thought to be caused by virus entry into vascular endothelial cells via the angiotensin-converting enzyme 2 receptor, which disrupts the intercellular junction, basal membrane, complement pathway, cytokine formation, and fibrin deposition.^12^

Research on hemostasis laboratory parameters during admission or the assessment of COVID-19 patients is mandated to predict the management of coagulopathy. Early and precise predictor variables based on hemostasis laboratory results, especially D-dimer, are also critical for identifying the risk and survival of COVID-19 patients as D-dimer has been strongly suggested as a marker of hypercoagulability due to its formation from fibrin formation and fibrinolysis.^13,14^ The objective of this research was to determine the relationship between D-dimer levels and the survival rate of COVID-19 patients.

## Materials and Methods

### Study Overview

This study was conducted in a retrospective cohort manner. This study took place at the Mohammad Hoesin General Hospital in Palembang, South Sumatera, Indonesia, specifically in the medical record and central laboratory department. From March to August 2020, we collected data on PCR-confirmed COVID-19 patients admitted to Mohammad Hoesin General Hospital. The study was approved by the institutional review board of Mohammad Hoesin General Hospital Palembang (Approval Number: 67/kepkrsmh/2020).

### Data Collection

Demographic, coexisting disorder, laboratory (on admission), and outcome data were obtained using electronic medical records from the hospitalized patients. The variables in this study were hematology and hemostasis results. The hematological parameters were leukocyte count (×10^9^/L), lymphocyte count (×10^9^/L), neutrophil count (×10^9^/), and neutrophil-lymphocyte ratio. The hemostasis parameters were prothrombin time (PT) [second], activated partial thromboplastin time (aPTT) [second], fibrinogen (g/L), and D-dimer (mg/L). All parameters that we collected were on admission result reports of in-patients in Dr. Mohammad Hoesin Hospital. The hematology test was performed on the Sysmex XN-1000, while the hemostasis test was conducted on the STA Compact Max-Stago. The immunoturbidimetric method was applied to perform the D-dimer test.

Patients have to be at least 18 years old, have complete data on hematological and hemostasis laboratory results, and also have complete medical records to be included. Patients on anticoagulants and thrombolytic agents, as well as those with bleeding disorders or cancer, were excluded from the study. The data on hemostasis (PT, aPTT, fibrinogen, and D-dimer) and hematological (white cell count, lymphocyte count, neutrophil count, and neutrophil-lymphocyte ratio) parameters were solely observed from the Mohammad Hoesin Hospital laboratory.

### Statistical Analysis

For continuous variables, the data were presented as median (minimum-maximum) due to abnormal data distribution. Categorical variables, on the other hand, are represented by the n (%) notation. The continuous data (age and all laboratory parameters) and categorical data (age,^15^ sex, severity, D-dimer) between non-survivor and survivor group were analyzed by using Mann–Whitney and Chi-square test (or Fisher’s exact when the expected count of <5 was more than 20%). The receiver operator characteristic (ROC) was used to determine the optimal D-dimer cutoff point. The Kaplan–Meier method was used to assess the prognosis (survival analysis). We were assessing the mortality as the outcome and D-dimer level as its predictor. Multivariable analysis was done using the Cox regression. The 95% CI and the hazard ratio (HR) were provided. Log-rank test was used to determine the average difference in survival time. The adjusted survival curve was generated for the covariates from a Cox proportional hazard model. Youden index was calculated to determine the optimal cut-off value (1.49 mg/L), with 50% of the patients still surviving for 30 days of admission. The *P*-value of <.05 (two-tailed) was considered statistically significant. The power analysis of 287 samples was shown a sufficient result for the prediction of mortality, with >90% of statistical power. MedCalc for Windows, version 19.3 (MedCalc Software, Ostend, Belgium), International Business Machines Statistical Package for Social Sciences (SPSS^®^), version 25.0 (IBM SPSS Corp., Armonk, NY, USA), and STATA version 15 (StataCorp LLC, College Station, Tex, USA) software were used for the data analysis.

## Results

**Figure 1.**
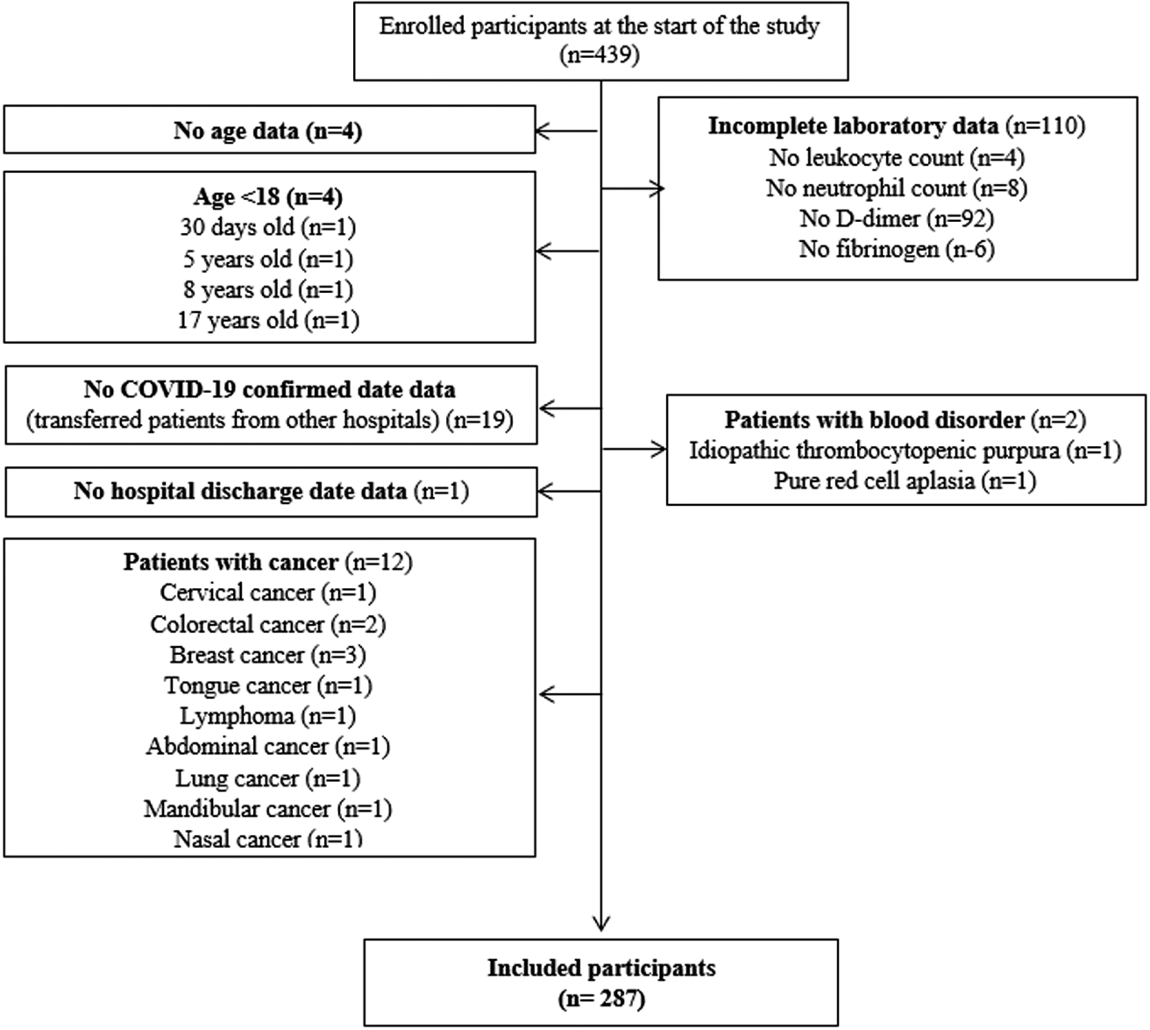
Study flow diagram on the numbers of participants enrolled and assessed during the study period.

From March to August 2020, we extracted the data on the admission of 439 COVID-19 patients who had a PCR-confirmed state in the onsite clinical laboratory. Following the completion of the inclusion criteria, 287 patients were enrolled in the study (Figure 1). Fifty-two people did not survive and 235 people did. Table 1 shows that there was a statistically significant difference between survivors and non-survivors in terms of demographic (age, sex) and laboratory (PT, fibrinogen, D-dimer, lymphocyte; neutrophil count, and neutrophil-lymphocyte ratio) parameters.

In demographic data analysis, the most essential factors were age and gender. In our study, the median age of non-survivors was higher than that of survivors (57.5 vs. 39.0 years). There was also a gender disparity, with men (73.1%) being more vulnerable to death. Meanwhile, in the survivor group, men made up 41.7% of the total population.

During this study, we also observed the laboratory results. Although the number is within the acceptable levels (12-18 s), the PT is significantly different (*P* = .010) between the survivor and non-survivor groups. The fibrinogen parameter showed similar results, but the average value was higher than the reference range (*P* < .001). Additionally, in those patients, a decreased level of lymphocyte (*P* < .001), a greater amount of neutrophil (*P* < .001), and an increase in neutrophil-lymphocyte ratio (NLR) value (*P* < .001) were all considered significant.

**High D-Dimer Levels May Be Predictive of a Poor Patient Outcome**

**Figure 2.**
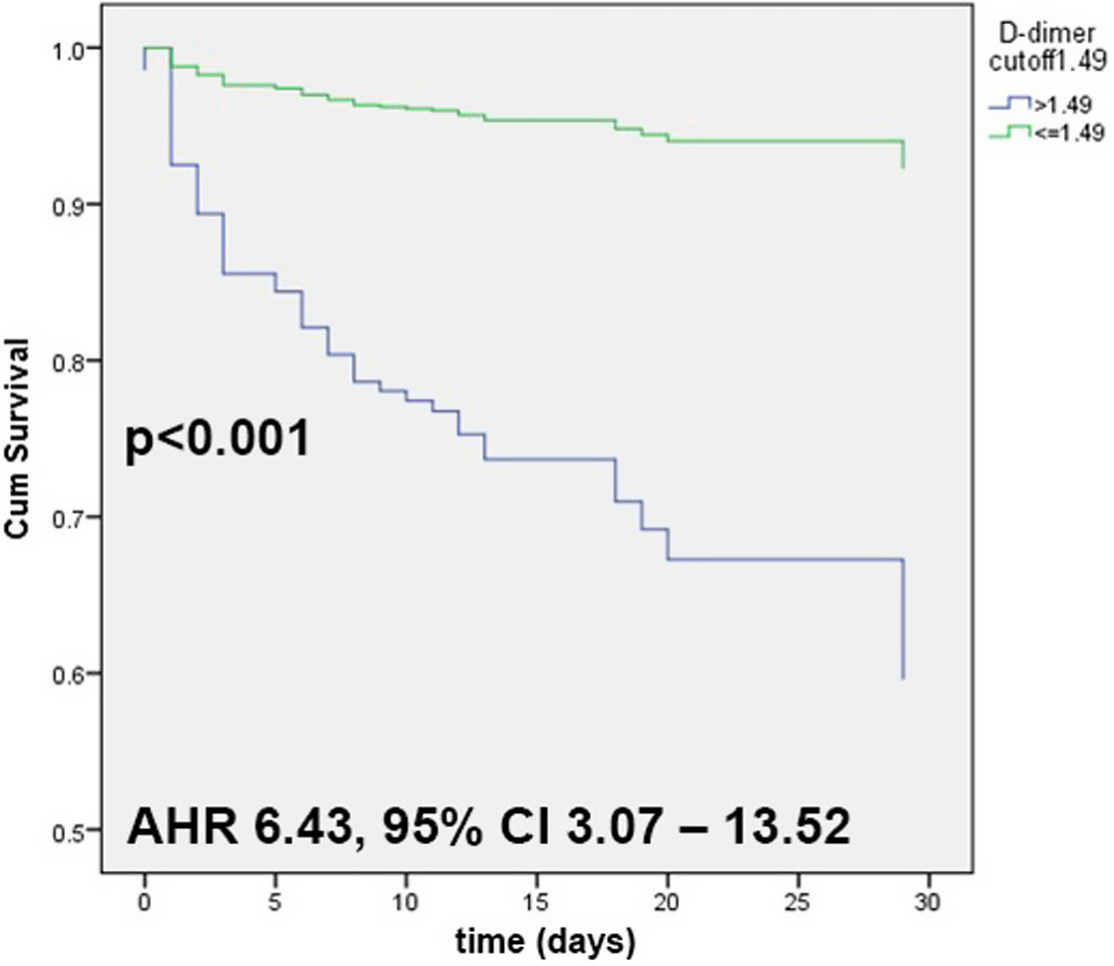
The adjusted Kaplan–Meier survival curves among COVID-19 patients. COVID-19, coronavirus disease 2019.

The D-dimer levels of 169 COVID-19 patients on admission were ≤1.49 mg/L, and 118 patients had D-dimer levels greater than 1.49 mg/L, according to the cut-off value. There were 52 non-survivors, with 43 having D-dimer levels >1.49 mg/L and the rest having D-dimer levels of ≤1.49 mg/L. Male patients had a lower cut-off for D-dimer in comparison with female patients (>1.49 mg/L vs. >2.2 mg/L). Figure 2 shows the sex and comorbidities-adjusted survival curve between the patients with D-dimer levels of >1.49 mg/L and less than or equal to 1.49 mg/L. The unadjusted Kaplan–Meier plot is also provided in Supplementary Figure 1. The ROC curve for D-dimer’s predictive role on patient death demonstrated 82.69% sensitivity and 68.09% specificity at the 1.49 mg/L cut-off. This graph’s area under curve (AUC) was 0.786 (*P* < .001).

Patients with D-dimer levels of >1.49 mg/L had a significantly higher risk of subsequent mortality (*P* < .001), as shown by the lower 30-day survival rate than patients with D-dimer levels of 1.49 mg/L, according to Kaplan-Meier curves (Figure 2), and were analyzed using the Mantel–Haenszel log-rank test. The difference in average survival time between these groups is about 8 days (29 vs. 21 days). During hospitalization, there were 52 death occurrences, 43 of which were observed in patients with D-dimer levels of >1.49 mg/L on admission, while only 9 in those with lower D-dimer levels (HR = 8.72, 95% CI: 4.24-17.93, *P* < .001). After the multivariable analysis, only the D-dimer, sex, and coexisting disorders were found to be significant determinants for the risk of COVID-19 mortality. The adjusted HR value was provided in Table 2. D-dimer showed the second-highest value of the C-index to predict in-hospital mortality in COVID-19 patients among regularly observed laboratory tests (Table 3).

## Discussion

The D-dimer antigen is a unique marker of fibrin degradation that may indicate infection-related coagulation effects.^14,16^ Critically ill patients with COVID-19 have extremely high D-dimer levels, which can lead to clotting disorders and peripheral microthrombi formation.^17^ In this study, we investigated the D-dimer levels on admission to see whether they could predict COVID-19 patient mortality.

Non-survivors were generally considered to be older in our investigation (57.5 vs. 39.0 years). During previous severe acute respiratory syndrome (SARS) and the Middle East respiratory syndrome (MERS) outbreaks, the same phenomenon has been observed,^18^ which is postulated to be associated with the decline of interferon-beta1 expression, a shortage of T and B cell function, and overproduction of type 2 cytokines as people get older. It was hypothesized that these events would result in inadequate control of viral replication and prolonged pro-inflammatory response.^10^ Men were also more probable to be non-survivors (73.1%). This is in line with a study by Zhou et al.^10^ which found a relatively similar rate (70%) of men in the non-survivors’ group. Attributed to the variations in the population affected by COVID-19, the data may vary.

The study’s main finding was that a D-dimer level of >1.49 mg/L on admission was an individual predictor of COVID-19 patient fatalities. It is based on the ROC curve (sensitivity: 82.69%, specificity: 68.09%). This finding provides a threshold value for identifying COVID-19 patients with a poor prognosis at an early stage. One hundred sixty-nine COVID-19 patients had D-dimer levels of ≤1.49 mg/L on admission, whereas 118 patients had D-dimer levels greater than 1.49 mg/L. There were 52 non-survivors, mostly belonging to the higher D-dimer group (43 vs. 9). A previous study by Zhang et al^7^ showed that the predominant D-dimer levels observed in their research were less than 2.00 mg/L on admission (276 patients), while 67 patients had D-dimer levels >2.00 mg/L. Thirteen deaths happened in their study, predominantly in the higher D-dimer level group (12 vs. 1). Increased D-dimer levels, as well as fibrin degradation products, have been commonly associated with coagulopathy, which can lead to thromboembolic events and devastating outcomes.^19,20^

D-dimer had the second-highest value in predicting in-hospital mortality, lower than the NLR, according to the C-index analysis. A previous study found that D-dimer predicts ICU admission less accurately than NLR (multivariable OR: 2.3 vs. 7.2, 95% CI, *P* < .001).^21^ However, a study of older (≥60 years) participants revealed that both D-dimer (AUC: 0.730, *P* < .001) and NLR (AUC: 0.715, *P* < .001) are equally accurate in predicting COVID-19 patients’ mortality.^22^ A study has shown that D-dimer has the highest C-index value in comparison with C-reactive protein, procalcitonin, and lactate dehydrogenase.^23^

According to the previous review by Abou-Ismail et.al,^24^ there are 3 different levels of D-dimer cut-off: 1.00 mg/L, 2.14 mg/L (sensitivity: 88.2%,specificity: 71.3%), and 2.00 mg/L (sensitivity: 92.3%, specificity: 83.3%). The distinction in the cut-off point value obtained in the present study with the research done by Zhang et al^7^ (1.49 mg/L vs. 2. 00 mg/L) could be due to the laboratory tools’ reading ability, which was only limited to 20.00 g/mL (10 patients had D-dimer levels of greater than 20.00 mg/L in our study), which may have influenced the cut-off results. The D-dimer level is highly sensitive (80%-100%) in detecting venous thromboembolism (VTE).^25,26^ They are not, however, specific for VTE because they can be elevated in sepsis, pregnancy, malignancy, and postoperative situations. This occurrence has been turned into a benefit in prognostication.^7^

The SARS-CoV-2 infection causes dysregulation of coagulation/anticoagulation cascades, which results in several pulmonary pathologies, including cellular fibromyxoid exudates, pneumocyte desquamation, hyaline membrane formation, hyaline membrane pulmonary edema, and interstitial inflammatory mononuclear cell infiltrates as seen in the previous SARS and MERS cases.^27-29^ One of the observed events, the elevated D-dimer levels are assumed to suggest a hyperfibrinolysis condition and increased inflammatory pressure triggered by SARS-CoV-2 infection.^30^

Increased D-dimers in COVID-19 patients demonstrate a hypercoagulable state caused by a variety of factors. The viral infection triggers a pro-inflammatory response, which is followed by an insufficient anti-inflammatory immune activity.^31^ Endothelial cell dysfunction may result from these events, resulting in excessive thrombin secretion. Hypoxia in severe COVID-19 patients may then increase blood viscosity and activate pathways that rely on hypoxia-induced transcription factors.^32^ The hospitalized patients, particularly those with severe COVID-19, are more likely to be older, have comorbidities, such as being in bed longer, and receive invasive treatments, all of which increase their risk of hypercoagulation or thrombosis.^33^ Additionally, several other patients may develop coagulopathy as a result of sepsis or disseminated intravascular coagulation.^20^

Regarding the lower cut-off points for the D-dimer in male patients observed in our study, it is in accordance with the previous study which is screening the VTE patients. It is found that the D-dimer level in those patients was lower in males as compared with females in the settings of the absence of pulmonary embolism.^34^ However, in COVID-19 patients, the level of D-dimer was found to be higher in males,^35^ although the proportion of abnormally high D-dimer level was not significantly different.

We realize that there are several limitations in this research, including coexisting disorders suffered by the patients (heart disease, metabolic disease), and some other intervention procedures done by doctors, all of which can cause bias in this study. The incomplete findings of laboratory and medical record data are also becoming a limitation of our study. Furthermore, not assessing treatment regimens is also a drawback. According to our findings, there is a risk of selection bias in this study because the survivor and non-survivor groups were unevenly distributed.

In conclusion, the D-dimer level is considered to be a sensitive predictor of COVID-19 patients’ in-hospital mortality. In our investigation, we discovered that D-dimer was the second best laboratory indicator for predicting patient mortality. As a result, the hospital may be able to lower death rates by paying closer attention to laboratory results, particularly D-dimer levels in COVID-19 patients. More research is needed to rule out confounding factors that could affect the relationship between D-dimer levels and COVID-19 mortality, making the connection more useful, as well as to monitor D-dimer levels in COVID-19 patients from admission to discharge.

## Figures and Tables

**Supplementary:**
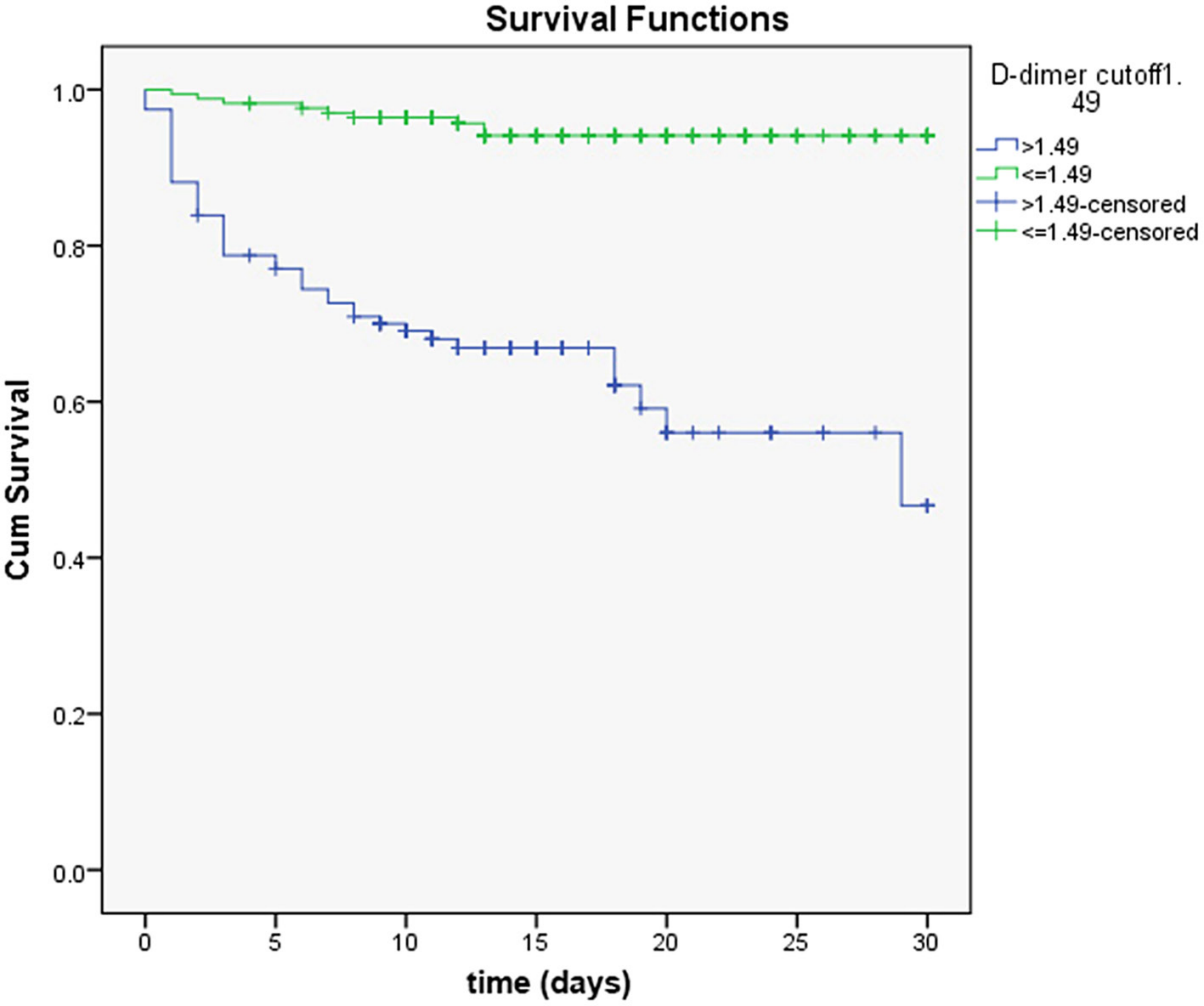
Figure 1

**Table 1.** Characteristics of COVID-19 Patients

Non-survivors (n = 52)	Survivors (n = 235)
Variable	Survival Rate COVID-19 Patients		*P*
	Age, years 15-47 48-63 ≥64	57.5 (29-77) 12 (23.1%) 23 (44.2%) 17 (32.7%)		39 (19-81) 145 (61.7%) 66 (28.1%) 24 (10.2%)	<.001^a^ <.001^b^
Sex			<.001^b^
Female	14 (26.9%)	137 (58.3%)	
Male	38 (73.1%)	98 (41.7%)	
Coexisting disorder	30 (57.7%)	51 (21.7%)	<.001^b^
Hypertension	9 (7.2%)	19 (8%)	.064^b^
Diabetes	12 (23.0%)	16 (6.7%)	.001^b^
Heart disease	3 (5.7%)	12 (5.0%)	.074^b^
Chronic kidney disease	4 (7.6%)	10 (4.2%)	.292^b^
Chronic liver disease	2 (3.8%)	1 (0.4%)	.083^b^
COPD	0 (0%)	1 (0.4%)	1.000^b^
Severity			<.001^b^
Severe	52 (100%)	5 (2.1%)	
Non-severe	0 (0%)	230 (97.9%)	
Hemostasis tests on admission			
PT, s	15.45 (11.40-53.10)	13.90 (11.50-57.40)	<.001^a^
n = 194			
<12	1 (2.6%)	2 (1.3%)	.067^b^
12-18	32 (84.2%)	148 (94.9%)	
>18	5 (13.2%)	6 (3.8%)	
aPTT, s	29.40 (20.80-48.00)	30.10 (21.20-53.20)	.608^a^
n = 194			
<27	10 (26.3%)	19 (12.2%)	.066^b^
27-42	26 (68.4%)	132 (84.6%)	
>42	2 (5.3%)	5 (3.2%)	
Fibrinogen, g/L	5.94 (1.11-12.00)	407(1.11-10.01)	<.001^a^
<2	1 (1.9%)	3 (1.3%)	<.001^b^
2-4	5 (9.6%)	110 (46.8%)	
>4	46 (88.5%)	122 (51.9%)	
D-dimer, mg/L	3.41 (0.44-20)	0.98 (0.09-20)	<.001^a^
>1.49 ≤1.49	43 (82.7%) 9 (17.3%)	75 (31.9%) 160 (68.1%)	<.001^b^
Hematologic tests on admission			
White-cell count, ×10^9^/L	12.335 (3.770-30.930)	8.410 (2.470-307.030)	<.001^a^
<4.730	3 (5.8%)	14 (6.0%)	<.001^b^
4.730-10.890	17 (32.7%)	158 (67.2%)	
>10.890	32 (61.5%)	63 (26.8%)	
Lymphocyte count, ×10^9^/L	1.103 (0.219-3.500)	1.845 (0.224-24.562)	<.001^a^
≤1.500	39 (75.0%)	77 (32.8%)	<.001^b^
>1.500	13 (25.0%)	158 (67.2%)	
Neutrophil count, ×10^9^/L	10.258 (2.865-25.053)	5.600 (0.804-34.527)	<.001^a^
≤4.695	6 (11.5%)	89 (37.9%)	<.001^b^
>4.695	46 (88.5%)	146 (62.1%)	
Neutrophil-lymphocyte ratio	9.56 (2.45-97.00)	3.05 (0.002-48.00)	<.001^a^
<3.13	4 (7.7%)	122 (51.9%)	<.001^b^
≥3.13	48 (92.3%)	113 (48.1%)	

Data are n (%), median (minimum–maximum). *P-*values were calculated by ^a^Mann–Whitney test or ^b^Chi-square/Fisher’s exact test, as appropriate.

COPD, chronic obstructive pulmonary disease; PT, prothrombin time; aPTT, activated partial thromboplastin time; COVID-19, coronavirus disease 2019.

**Table 2.** Adjusted Hazard Ratio (HR) of In-Hospital Mortality Among Patients with COVID-19 for D-Dimer, Sex, and Coexisting Disorders

Variable	Adjusted HR	95% CI	*P*
D-dimer ≤1.49 >1.49	1 (REF) 6.43	3.07-13.52	<.001
Sex Male Female	1 (REF) 2.86	1.55-5.29	.001
Coexisting disorders No Yes	1 (REF) 2.40	1.36-4.24	.002

**Table 3.** C-Statistic Analysis of Routine Laboratory Tests to Predict Mortality in COVID-19 Patients

Parameter**s**	C-index	95% CI
D-dimer	0.79	0.73-0.83
Neutrophil count	0.77	0.69-0.84
Lymphocyte count	0.25	0.18-0.32
Neutrophil-Lymphocyte ratio	0.83	0.78-0.88
White blood cells count	0.71	0.62-0.79
PT	0.71	0.61-0.81
aPTT	0.47	0.36-0.59

PT, prothrombin time; aPTT, activated partial thromboplastin time.
